# Severe maternal morbidity associated with maternal birthplace in three high-immigration settings

**DOI:** 10.1093/eurpub/cku230

**Published:** 2015-01-13

**Authors:** Marcelo L. Urquia, Richard H. Glazier, Laust Mortensen, Anne-Marie Nybo-Andersen, Rhonda Small, Mary-Ann Davey, Mattias Rööst, Birgitta Essén, for the ROAM (Reproductive Outcomes and Migration. An International Collaboration)

**Affiliations:** 1 St. Michael’s Hospital, Li Ka Shing Knowledge Institute, Dalla Lana School of Public Health, University of Toronto, Toronto, Canada; 2 Institute for Clinical Evaluative Sciences, Toronto, Canada; 3 Institute of Public Health, University of Copenhagen, Copenhagen, Denmark; 4 Mother and Child Health Research Centre, School of Nursing and Midwifery, LaTrobe University, Melbourne, Australia; 5 Department of Women’s and Children’s Health/International Maternal Child Health, Uppsala University, Uppsala, Sweden

## Abstract

**Background:** Maternal mortality and morbidity vary substantially worldwide. It is unknown if these geographic differences translate into disparities in severe maternal morbidity among immigrants from various world regions. We assessed disparities in severe maternal morbidity between immigrant women from various world regions giving birth in three high-immigration countries. **Methods:** We used population-based delivery data from Victoria; Australia and Ontario, Canada and national data from Denmark, in the most recent 10-year period ending in 2010 available to each participating centre. Each centre provided aggregate data according to standardized definitions of the outcome, maternal regions of birth and covariates for pooled analyses. We used random effects and stratified logistic regression to obtain odds ratios (ORs) with 95% confidence intervals (95% CIs), adjusted for maternal age, parity and comparability scores. **Results:** We retrieved 2,322,907 deliveries in all three receiving countries, of which 479,986 (21%) were to immigrant women. Compared with non-immigrants, only Sub-Saharan African women were consistently at higher risk of severe maternal morbidity in all three receiving countries (pooled adjusted OR: 1.67; 95% CI: 1.43, 1.95). In contrast, both Western and Eastern European immigrants had lower odds (OR: 0.82; 95% CI: 0.70, 0.96 and OR: 0.64; 95% CI: 0.49, 0.83, respectively). The most common diagnosis was severe pre-eclampsia followed by uterine rupture, which was more common among Sub-Saharan Africans in all three settings. **Conclusions:** Immigrant women from Sub-Saharan Africa have higher rates of severe maternal morbidity. Other immigrant groups had similar or lower rates than the majority locally born populations.

## Introduction

Maternal mortality and morbidity vary substantially worldwide, with poor countries having the highest risks.^1^^,^^2^ Virtually all Western developed countries have become high-immigration countries and increasingly receive migrants from middle and low-income countries,^3^^,^^4^ where the incidence of maternal morbidity and mortality is high.^2^^,^^5^ Although some pregnancy complications such as gestational diabetes^6^ or pre-eclampsia^7^ are known vary according to immigrants’ country of birth, less is known about rarer life-threatening conditions (e.g., uterine rupture, sepsis) among immigrants to industrialized countries, particularly what world regions of origin are associated with higher and lower risk.

Severe maternal illness or ‘near miss’ conditions are increasingly used as indicators of quality of maternal care, to investigate risk factors for the occurrence of disease and progression to death, and to inform surveillance systems, service providers and policy makers in the area of maternal health.^1^^,^^8–11^ The few previous studies found an overall increased risk of severe maternal morbidity among ‘non-western’ vs. ‘Western women’ in the Netherlands,^12^ ‘non-white’ groups of women vs. ‘white’ women in the UK,^13^^,^^14^ women of ‘non-Italian nationality’ vs. nationals^15^ and women from ‘low income countries’ vs. Swedish-born.^16^ Comparability and generalizability of findings are compromised by different study populations, outcome definitions and diverse and mixed criteria to classify foreign groups (e.g., self-reported ethnicity, woman’s country of birth including her parents’ country of birth and nationality) as well as different circumstances of health care.^17^

The aim of this study was to assess differences in severe maternal morbidity among immigrants residing in three high-immigration settings according to their maternal region of birth with the purpose of providing a more robust approach to identifying maternal regions of origin associated with higher (and lower) global risk of severe maternal morbidity in receiving countries. In this study, we focus on maternal world regions of birth, since maternal country of birth has been identified as one key predictor of immigrants’ perinatal health,^18^ reflecting early childhood exposures and background risk factors acquired in the source countries. Comparability between receiving countries is strengthened by the fact that the three study settings (Victoria, Australia; Ontario, Canada and Denmark) provide basic universal health care services to pregnant women, and receive large number of immigrants, many of which are admitted under humanitarian grounds. We also used standardized definitions of the outcome and immigrant groups across countries. We compared outcomes of immigrants from the main regions of the world to those of women born in three receiving settings.

## Methods

### Study design

This is a cross-country comparative study. We used population-based regional or national health data from three countries (Australia, Canada and Denmark). The study protocol was specified prior to data collection. Participating centres had to meet the following inclusion criteria: (i) ascertainment of severe maternal morbidity adapted from a recent study in Sweden [coded according to by the International Statistical Classification of Diseases and Related Health Problems,10th Revision (ICD-10)^19^], (ii) categorization of maternal region of origin according to pre-specified groupings by maternal country of birth; (iii) provide population-based aggregated data (number of cases and non-cases) stratified by maternal region of birth, parity and maternal age groups.

### Study populations and data sources

The study population was composed of all women giving birth (including live births and stillbirths), both immigrants and non-immigrants occurring in the most recent 10-year period available to each participating centre (table 1). The unit of analysis was the delivery. We included deliveries from Australia (the state of Victoria), Canada (province of Ontario) and Denmark (national data). Population health data were obtained from perinatal data collections, health care registers and birth certificates linked to hospital records. These databases have been previously used for perinatal research and have been found to be of good quality.^8^^,^^20–23^ Each participating centre was responsible for assessing the internal consistency and quality of the definitions in the datasets across years and jurisdictions and to obtain ethical approval to share the data for pooled analyses. The study protocol was approved by the Research Ethics Board of the St. Michael’s Hospital, Toronto, Canada.

**Table 1:** Characteristics of the study populations and datasets

	Victoria, Australia	Ontario, Canada	Denmark
Total number of deliveries	636,042	1,050,688	636,177
Period	01/01/1999 to 31/12/2008	01/04/2002 to 31/03/2010	01/01/2000 to 31/12/2009
Inclusion criteria of delivery episodes	All deliveries to women who gave birth at 20 or more weeks’ gestation (or if gestation not known ≥400 g)	All in-hospital deliveries resulting in liveborns and stillborns	All deliveries resulting in liveborns and stillborns ≥ 28 weeks 2000-3 and ≥ 22 weeks 2004-9
Source	VPDC (A)	DAD (B), CIC (C)	DCRS (D), DNPR (E)
Linkage method	Not linked—all data reported by attending mid-wife	Probabilistic	Deterministic
ICD version	ICD-10	ICD-10	ICD-10
No of diagnosis fields	23	25	No limit
Maternal country of birth	Country of birth (self-reported)	Country of birth (register-based)	Country of birth (register-based)

Abbreviations: (A) Victorian Perinatal Data Collection, Victoria, Australia; (B) Discharge Abstracts Database, Canadian Institute for Health Information, Canada; (C) Citizenship and Immigration Canada Database; (D) Danish Civil Registration System, The Ministry of Social Affairs and Integration; (E) Danish National Patient Register, Danish National Board of Health.

### Outcome measure

We defined severe maternal morbidity as the occurrence of one or more of a list of life threatening complications that occurred during pregnancy or delivery and were recorded in the index delivery hospitalization, based on a set of ICD-10 clinical diagnostic codes used in a national Swedish study.^16^ Conditions included organ failure, shock, severe pre-eclampsia, uterine rupture and sepsis, among others listed in Supplementary table 1 (URL). Unlike the Swedish study, we could not include intervention codes due to the lack of comparability between countries.

### Maternal region of birth

Information on maternal country of birth was used to create maternal world regions of origin, based on the United Nations sub-regions.^24^ Groups were as follows: Eastern Europe, Western Europe, Latin America and the Caribbean, North Africa and Middle East, Sub Saharan Africa, South Asia and East-Southeast of Asia. North America and Oceania were not included because of small numbers of immigrants from these regions. The reference group was composed of the receiving country-born; Australian-born in Australia, and so on, to assess how different immigrant groups fare against the non-immigrant populations of the respective receiving countries.

### Covariates

We included maternal age and parity as potential confounders. Because maternal age has a J- or U-shape association with severe maternal morbidity,^8^^,^^25^ we created two groupings to capture the excess risk associated with the extremes of the maternal age distribution: <20 years or ≥35 years vs. 20-34 years as the reference group. Parity was dichotomized in primiparous vs. multiparous women as the reference group. Inclusion of additional covariates or more detailed strata of maternal age and parity were not feasible due to restrictions on disclosing small cell sizes in Ontario, Canada and Victoria, Australia.

For secondary analyses restricted to Ontario data, we further included in the multivariable models multiple pregnancies (yes, no), marital status (single, married or common-law and separated, divorced or widowed), maternal education (no high school diploma, high school diploma, some post-secondary education and university diploma) and knowledge of official Canadian languages at arrival (English, French, both, neither), refugee status (yes, no), neighbourhood income (quintiles), and duration of residence in Canada (in years).

### Statistical analyses

Count data (number of cases and non-cases of severe maternal morbidity) were provided by each participating centre by strata of maternal region of birth, maternal age and parity. These count data were merged into a single dataset with the addition of a receiving country identifier. The resulting dataset was then imported into and analysed with SAS 9.3 (SAS Institute, Cary, NC). Outcome rates were expressed in number of cases per 1000 deliveries.

We performed two analyses. First, we compared different immigrant groups with the non-immigrant population within each receiving country using stratified logistic regression. Regression models were run before and after adjustment for maternal age and parity. Effect estimates were odds ratios (ORs) with 95% confidence intervals (CIs).

Second, we used a random effects model with deliveries (first level) nested within receiving countries (second level). The model included random intercepts to account for the variability of the outcome between receiving countries. This model is equivalent to a random effects meta-analysis in which each country represents a ‘study’ and provides a summary (pooled) estimate of the effects of each maternal world region across receiving countries. To account for potential variations in circumstances of health care between receiving countries (i.e., geographic setting, health care setting, type of health care providers and differences in the medical management between comparison groups) we further adjusted for a recently developed comparability score^17^ by assigning a weight of 1/8 to each observation of the reference groups (native-born) in each receiving setting. Giving less weight to the reference group is equivalent to reducing the sample size and power to detect statistical significant associations, thus penalizing for each dimension not directly comparable between groups. Because the use of comparability score may also result in type II error, we report both the associations before and after applying the comparability score.

Third, we reported the ranking and percentage of cases affected by the top three diagnoses, by women’s birthplace and place of delivery.

Lastly, we conducted secondary analyses restricted to Ontario data to assess (i) the extent of residual confounding due to unmeasured covariates in the main analysis, by further controlling for a wide array of sociodemographic and immigrant characteristics listed earlier, and (ii) whether the risk of severe maternal morbidity differed between Latin American women vs. those from the Caribbean, who were classified in the same group in the main analysis.

## Results

There were 2,322,907 deliveries across the three receiving countries, 479,986 (20.7%) of which were to immigrant women (table 2). The occurrence of any severe maternal morbidity varied from 2.1 per 1000 deliveries in Victoria, Australia to 4.8 cases per 1000 deliveries in Denmark.

**Table 2:** Characteristics of the study population, by place of residence at delivery^a^

	Place of residence at delivery		
	Victoria, Australia	Ontario, Canada	Denmark
Total number of deliveries	636,042	1,050,688	636,177
Cases of severe maternal morbidity (rate per 1,000 deliveries)	1,316 (2.1)	3,062 (2.9)	3,085 (4.8)
Maternal characteristics			
Maternal age <20 years or ≥35 years	160,151 (25.2)	253,053 (24.1)	122,092 (19.2)
Nulliparity	270,937 (42.6)	477,701 (45.6)	273,956 (43.1)
Region of origin			
Receiving country-born	497,113 (78.2)	789,581 (75.2)	556,227 (87.4)
Immigrants (all)	138,929 (21.8)	261,107 (24.8)	79,950 (12.6)
Immigrants (by region of birth)			
Western Europe	35,288 (5.5)	17,190 (1.6)	19,794 (3.1)
Eastern Europe	4,760 (0.8)	19,682 (1.9)	5,503 (0.9)
Latin America and the Caribbean	4,027 (0.6)	36,197 (3.5)	1,989 (0.3)
South Asia	17,625 (2.8)	87,505 (8.3)	10,057 (1.6)
East-Southeast Asia	49,150 (7.7)	59,719 (5.7)	8,324 (1.3)
North Africa and Middle East	18,005 (2.8)	21,361 (2.0)	25,066 (3.9)
Sub Saharan Africa	10,074 (1.6)	19,453 (1.8)	9,217 (1.5)

^a^Expressed as number (percent) unless otherwise specified.

The proportion of deliveries to immigrant women varied across countries, from 12.6% in Denmark to 24.8% in the Ontario. The proportion of women of extreme maternal age ranged from 19.2% in Denmark to 25.2% in Victoria. There was little variation in the proportion of primiparous women between countries. The distribution of deliveries by maternal birthplace also varied markedly across countries, reflecting different migration patterns.

Table 3 shows the results of separate ordinary logistic regression analyses conducted within strata of receiving country. Compared with receiving country-born women, those from Sub-Saharan Africa were the only group at higher risk in all three countries. These disparities ranged from an OR = 1.50 in Ontario to OR = 2.00 in Victoria, probably reflecting differences in the baseline risk of the local populations (reference groups). In Victoria, no other groups differed from native-born women with regard to risk of severe maternal morbidity. In Ontario, most immigrant groups fared better than the majority population, with the exceptions of women from Sub-Saharan Africa, who fared worse, and those from Latin America and the Caribbean, who showed no difference. Women from East-Southeast Asia were the only group with lower rates of severe maternal morbidity than the native-born population in Denmark.

**Table 3:** ORs with 95% CIs of the association between maternal region of birth and severe maternal morbidity, by receiving country and all three receiving countries combined

	Victoria, Australia (*N* = 636,042)		Ontario, Canada (*N* = 1,050,688)		Denmark (*N* = 636,177)		Pooled (*N* = 2,322,907)	
Maternal birthplace	Rate per 1,000	AOR^a^ (95% CI)	Rate per 1,000	AOR^a^ (95% CI)	Rate per 1,000	AOR^a^ (95% CI)	AOR^b^ (95% CI)	AOR^c^ (95% CI)
Born in receiving country	2.0	1.00	3.0	1.00	4.9	1.00	1.00	1.00
Western Europe	2.1	1.02 (0.80, 1.29)	2.2	0.70 (0.51, 0.97)	4.1	0.83 (0.67, 1.04)	0.85 (0.74, 0.98)	0.82 (0.70, 0.96)
Eastern Europe	1.9	0.94 (0.49, 1.82)	1.4	0.45 (0.31, 0.65)	4.4	0.88 (0.59, 1.31)	0.62 (0.48, 0.80)	0.64 (0.49, 0.83)
Latin America and Caribbean	2.2	1.11 (0.58, 2.15)	3.2	1.04 (0.87, 1.26)	6.5	1.30 (0.75, 2.24)	1.09 (0.92, 1.29)	1.13 (0.94, 1.37)
South Asia	2.5	1.27 (0.94, 1.71)	2.3	0.79 (0.68, 0.91)	4.4	0.91 (0.67, 1.23)	0.87 (0.77, 0.98)	0.89 (0.78, 1.03)
East-Southeast Asia	2.3	1.14 (0.94, 1.38)	2.4	0.76 (0.64, 0.90)	3.0	0.61 (0.41, 0.90)	0.85 (0.76, 0.96)	0.85 (0.74, 0.97)
North Africa and Middle East	2.1	1.12 (0.81, 1.55)	2.2	0.73 (0.54, 0.98)	4.1	0.86 (0.70, 1.04)	0.86 (0.75, 1.00)	0.85 (0.72, 1.00)
Sub-Saharan Africa	3.9	2.00 (1.45, 2.75)	4.5	1.50 (1.21, 1.85)	8.5	1.76 (1.40, 2.21)	1.68 (1.46, 1.93)	1.67 (1.43, 1.95)

Abbreviations: AOR = adjusted odds ratio, CI = confidence interval. ^a^Obtained with stratified logistic regression models, adjusted for maternal age and parity. ^b^Random effects model (receiving countries as random intercepts) adjusted for maternal age and parity. ^c^Random effects model (receiving countries as random intercepts) adjusted for maternal age and parity and further adjusted for comparability scores.

Pooled analyses combining data from all three countries, with deliveries as first level units and receiving countries as second level units, showed that, compared with the receiving country-born, women from Sub-Saharan Africa were at higher odds of severe maternal morbidity [adjusted odds ratio (AOR): 1.68: 95% CI: 1.46, 1.93]. European women had lower odds than receiving country-born women. These associations did not substantially change after adjusting for the comparability score, with the exception of a protective effect among South Asians, which was no longer statistically significant after applying the scores. Lower odds were also found for the two groups of Asia and women from North Africa and the Middle East, although these associations were of borderline statistical significance.

Table 4 ranks the top three diagnoses of the severe maternal morbidity indicator by immigrants’ birthplace. Severe pre-eclampsia was the most common diagnosis for most groups, with the only exception of Sub-Saharan African women, who were mostly affected by uterine rupture in all three countries.

**Table 4:** Proportional morbidity (and ranking) of the three top contributing diagnoses among cases of the composite severe maternal morbidity indicator, by region of birth and place of residence at delivery

Region of birth	Place of residence at delivery	Pre-eclampsia, incl HELLP syndrome or DIC		Uterine rupture		Cardiovascular diseases	
		*N* (%)	Rank	*N* (%)	Rank	*N* (%)	Rank
Born in receiving country	Victoria, AU (*N* = 990)	378 (40.2)	1	168 (17.9)	2	51 (5.4)	3
	Denmark (*N* = 2717)	1,342 (49.4)	1	801 (29.5)	2	293 (10.8)	3
	Ontario, CA (*N* = 2405)	897 (37.3)	1	745 (31.0)	2	341 (14.2)	3
Western Europe	Victoria, AU (*N* = 74)	29 (40.8)	1	15 (21.1)	2	-	-
	Denmark (*N* = 81)	30 (37.0)	1	28 (34.6)	2	13 (16.0)	3
	Ontario, CA (*N* = 37)	11 (29.7)	2	13 (35.1)	1	6 (16.2)	3
Eastern Europe	Victoria, AU (*N* = 9)	-	^a^	-	^a^	-	^a^
	Denmark (*N* = 24)	12 (50.0)	1	6 (25.0)	2	-	^a^
	Ontario, CA (*N* = 27)	8 (29.6)	1	8 (29.6)	1	-	^a^
Latin America and Caribbean	Victoria, AU (*N* = 9)	-	^a^	-	^a^	-	^a^
	Denmark (*N* = 13)	-	^a^	-	^a^	-	^a^
	Ontario, CA (*N* = 115)	50 (43.5)	1	20 (17.4)	2	11 (9.6)	4^b^
South Asia	Victoria, AU (*N* = 44)	21 (47.7)	1	11 (25.0)	2	-	^c^
	Denmark (*N* = 44)	14 (31.8)	1	12 (27.3)	2	9 (20.5)	3
	Ontario, CA (*N* = 202)	80 (39.6)	1	60 (29.7)	2	20 (9.9)	3
Rest of Asia/East-Southeast Asia	Victoria, AU (*N* = 113)	44 (40.7)	1	22 (20.4)	2	-	^c^
	Denmark (*N* = 25)	8 (32.0)	1	6 (24.0)	2	5 (20.0)	3
	Ontario, CA (*N* = 143)	55 (38.5)	1	30 (21.0)	2	21 (14.7)	3
North Africa and Middle East	Victoria, AU (*N* = 38)	16 (44.4)	1	8 (22.2)	2		
	Denmark (*N* = 103)	28 (27.2)	1	38 (36.9)	2	14 (13.6)	3
	Ontario, CA (*N* = 46)	13 (28.3)	1	19 (41.3)	2	8 (17.4)	3
Sub-Saharan Africa	Victoria, AU (*N* = 39)	13 (35.1)	2	17 (45.9)	1		
	Denmark (*N* = 78)	27 (34.6)	2	34 (43.6)	1	6 (7.7)	3
	Ontario, CA (*N* = 87)	28 (32.2)	2	31 (35.6)	1	9 (10.3)	3

Abbreviations: HELLP: HELLP syndrome (haemolysis, elevated liver enzymes, low platelet count); DIC: disseminated intravascular coagulation. ^a^Frequencies lower than five cases were suppressed. ^b^The third diagnosis for Latin Americans and Caribbean women in Ontario was Sepsis, with 15 cases (13.0%). ^c^The third diagnosis for South Asian and East-Southeast Asian women in Victoria, Australia, was Pulmonary Edema, with six cases (13.6%) and 13 cases (12.0%), respectively.

Supplementary table 2 (URL) describes the results of secondary analyses with the same Ontario data but now including additional control variables, such as immigration characteristics. Further adjustment did not substantially change the associations between maternal region of birth and severe maternal morbidity, suggesting that the observed associations in the main analysis are not seriously confounded by unmeasured factors like sociodemographics and immigration characteristics. When Latin American women were separated from Caribbean women, we did not find a statistically significant difference in their odds of the outcome (not shown), and therefore we kept them together. Interestingly, the relative disparities between immigrant groups increased when analyses were restricted to immigrants in Ontario and the reference group was composed of Western European immigrants. Compared with immigrants from Western Europe, Sub-Saharan Africans had twice the odds of severe maternal morbidity (AOR: 2.01; 95% CI: 1.35, 2.98) followed by women from Latin America and the Caribbean (AOR: 1.47; 95% CI: 1.01, 2.14).

## Discussion

### Main findings

Our main finding is that women from Sub-Saharan Africa had consistently the highest incidence of severe maternal morbidity in all three participating countries. The most frequent diagnosis among Sub-Saharan African women was uterine rupture, whereas other groups were mostly affected by severe pre-eclampsia. In contrast, the remaining maternal regions of birth were not associated with increased odds of severe maternal morbidity, with the possible exception of women from Latin American and the Caribbean. The rest of the immigrant groups were at lower risk, particularly Europeans. The non-immigrant populations generally had incidence rates situated somewhere in the middle of the continuum of risk.

### Strengths and limitations

Strengths are the use of large population-based datasets, the combination of data collected from three high-immigration countries and the use of a standardized definition of the outcome based on ICD-10 diagnostic codes and migrant groups.

Limitations are also several and illustrate the challenges faced by cross-country comparative studies. Although we used a common protocol for data collection, inclusion criteria varied somewhat between countries. We relied on perinatal data collections, health care registers and linked databases from different countries, which were not designed for research purposes and their differences may also impact on the absolute rates of the outcomes. Indeed, the rates of severe maternal morbidity were comparable in Victoria, Australia (2.1 per 1000 deliveries) and Ontario, Canada (2.9 per 1000 deliveries), but the rate was higher in Denmark (4.8 per 1000 deliveries). These differences may reflect different registration practices rather than higher morbidity among immigrants in Denmark, since the overall rates are mainly driven by the majority non-immigrant populations. The higher overall rate in Denmark may also suggest a higher baseline risk in the non-immigrant population, which, if true, could dilute the excess risk found among Sub-Saharan women in Denmark. However, as our analyses are based on the relative disparities according to maternal region of birth variations in absolute rates can be considered of less concern, assuming that the probability of being diagnosed does not differ between immigrants and non-immigrants. This assumption may not hold true if, for example, immigrants face more challenges with language in a non-English-speaking country such as Denmark than in Canada or Australia. Greater language barriers in one country may hamper the ability of early detection and contribute to progression to more severe complications. Nonetheless, the relative disparities were not higher in Denmark compared with Victoria and Ontario. Unfortunately, we could not account for all sources of variations between countries. We used comparability scores to compensate for unmeasured sources of variation between countries and the results did not substantially change.

Although we controlled for extreme maternal age and parity we could not include other covariates such as multiple pregnancies, history of morbidity and socio-economic indicators or health behaviours, due to either lack of information or restrictions on reporting low event counts in some countries. However, adjustment for multiple pregnancies, socio-economic indicators and various immigration characteristics in our secondary analyses using Ontario data did not substantially change the associations observed in the main analysis, suggesting that residual confounding due to unmeasured covariates is rather small.

The ability to create more detailed groupings of maternal origin, ideally at the country level, was hampered by sample size considerations and consistency across participating countries. Different pool of migrants may be responsible for part of the differences observed between receiving countries. National data were only available for Denmark and therefore the findings for Ontario and Victoria, although regionally valid, may not necessarily be representative of their respective countries as a whole.

### Interpretation

There are important variations in severe maternal morbidity among immigrants to industrialized countries according to their maternal birthplace The excess risk found among Sub-Saharan Africans is consistent with data from other countries, such as Belgium,^26^ Italy,^27^ the Netherlands^12^ and the UK.^13^^,^^14^ This pervasive pattern may be a reflection of the background risk in the source African countries. In fact, Sub-Saharan Africans exhibit the highest rates of pre-eclampsia, eclampsia^28^ and maternal mortality worldwide,^2^ and so it is not surprising that migrants may carry over their higher susceptibility post-migration. Spanish studies have linked higher maternal mortality with foreign-born women, particularly those of Sub-Saharan nationalities.^29^^,^^30^ A previous study found increased risk of caesarean section, stillbirth and low Apgar score among Somali women compared with native-born women in six countries, including Australia and Canada.^31^ Although the proportion of deliveries to Somali women in the Sub-Saharan Africa group was sizeable (21.2% in Victoria, 25.0% in Ontario and 69.3% in Denmark) it is likely that women from other African countries may be at higher risk too.

Another interesting finding is the predominance of uterine rupture among Sub-Saharan African women in all three countries, as opposed to severe pre-eclampsia, which was most common for the rest of women. However, more research is needed to fully understand this finding since the validity of the registration of uterine rupture has been shown to be questionable in a recent Danish study.^32^

The odds of severe maternal morbidity for Sub-Saharan African women relative to the local-born populations ranged from 1.50 in Ontario to 2.00 in Victoria. These differences, however, may be reflecting differences in the baseline risk of the local populations (reference groups) rather than worse health among Sub-Saharan Africans in Victoria compared with Ontario. In fact, Ontario is home of the largest black population in Canada and many Canadian-born women living in Ontario are of non-European descent,^33^ which could have diluted the associations. In addition, Ontario data misclassify a small proportion of immigrants as non-immigrants, further diluting the comparisons with the native-born group.

It is interesting to note that these disparities observed within and across countries, although sizeable, are not as large as the disparities observed between countries worldwide.^2^ This suggests that maternal country of birth may not accurately reflect the risks observed in the source countries and predict the actual risks experienced post-migration. Immigrants to industrialized countries may not be representative of their source populations, since many may be selected or self-selected on the basis of attributes associated with good health (i.e., healthy migrant effect),^34^ thus flattening the disparities. In addition, higher quality of care in industrialized countries may be also responsible for attenuating the disparities.

Latin American and Caribbean women were at somewhat higher risk of severe maternal morbidity, particularly in Denmark, although the association did not reach statistical significance. Most immigrants in this group in Denmark originate from South and Central America. Ontario analyses suggest that Latin American women do not substantially differ from Caribbean women in their risk of severe maternal morbidity.

The immigrant groups from Europe had lower incidence than the non-immigrant populations; this pattern being consistent with the healthy migrant effect.^34^ It is also possible to speculate that European immigrants may be more likely than others to travel and deliver in their places of origin, particularly when facing complications of delivery, thus deflating the numerators. This phenomenon is known as the salmon bias.^34^ Similar or lower rates of severe maternal morbidity among immigrants from Asia, North Africa and the Middle East may be also reflecting selective migration, but not necessarily salmon bias.

To sum up, our findings provide robust evidence of a high global risk of severe maternal morbidity among Sub-Saharan African immigrants in high-immigration settings of three continents. Identifying the causes of their excess risk may help provide Sub-Saharan African women enhanced surveillance and culturally sensitive antenatal and peripartum care.

## Funding

The study was possible thanks to the Institute for Clinical Evaluative Sciences (ICES), which is supported by an annual grant from the Ontario Ministry of Health and Long-Term Care. The opinions, results, and conclusions reported in this article are those of the authors and are independent from the funding sources. No endorsement by ICES or the Ontario Ministry of Health and Long-Term Care is intended or should be inferred. The study was partially funded by a grant of the Canadian Institutes of Health Research (MOP-123267). Marcelo Urquia holds a Canadian Institutes of Health Research New Investigator Award.

*Conflicts of interest*: None declared.

**Key points**

- We conducted a study of severe maternal morbidity among immigrants from different world regions giving birth in Australia, Canada and Denmark, using standardized definitions of the outcome and immigrant groups.
- Compared with non-immigrants, only Sub-Saharan African women were consistently at higher risk of severe maternal morbidity in all three receiving countries.
- Other immigrant groups had similar or lower rates than the majority locally born populations.
- Severe pre-eclampsia was the most common diagnosis, with the exception of Sub-Saharan African women, who were mostly affected by uterine rupture in all three countries.

## Supplementary data

Supplementary data are available at *EURPUB* online.
